# Clinical changes in the severity of dental fluorosis: a longitudinal evaluation

**DOI:** 10.1186/s12903-021-01729-3

**Published:** 2021-07-22

**Authors:** Alexandra Saldarriaga, Diego F. Rojas-Gualdrón, Manuel Restrepo, Diego Girotto Bussaneli, Camila Fragelli, Rita de Cássia Loiola Cordeiro, Lourdes Santos-Pinto, Fabiano Jeremias

**Affiliations:** 1 Department of Pediatric Dentistry and Orthodontics, São Paulo State University (Unesp), Araraquara School of Dentistry, Rua Humaitá, 1680, Araraquara, SP 14801-903 Brazil; 2 Research Department, School of Dentistry, CES University, Medellín, Colombia; 3 School of Medicine, CES University, Medellín, Colombia

**Keywords:** Dental fluorosis, Permanent dentition, Severity, Longitudinal

## Abstract

**Background:**

Dental fluorosis (DF) has been one of the most prevalent pediatric dental conditions associated with aesthetic concern and treatment needs. This study aimed to identify the longitudinal clinical change in the severity of DF in 8–12-year-old children and its association with gender, age, severity, and tooth type.

**Methods:**

This observational study assessed the dental aspects of the 92 Colombian children in 2015 (mean age at beginning 9.71 years ± 1.23) and 2018 (mean age 13.69 years ± 1.41), from an area with high DF prevalence. DF was recorded in all permanent teeth by two calibrated examiners using the Thylstrup and Fejerskov Index (TFI). DF severity change (maximum-TFI-score) was analyzed with descriptive analysis at the tooth level. Associated factors were evaluated with the generalized linear model, binomial family, and logarithmic link function.

**Results:**

TFI scores ranged between 1 (very mild) to 6 (severe), being score 2 (41.7%) the most prevalent. After three years, 29.6% of the teeth presented score reduction, 24.1%, increased and 46.3% did not change; the significant association was related to increasing of the basal TFI = 1 score (44.2%) (RR = 9.7; 95% CI 1.7–56.5; p = 0.01) and with canines, premolars and second-permanent-molars teeth group (RR = 3.3; 95% CI 1.9–5.6; p = 0.005).

**Conclusion:**

The present study based on clinical features about DF confirms the dynamic post-eruptive nature of this condition. After three years of follow-up, a considerable proportion of the teeth changed to a higher score. Furthermore, the canines, premolars, and second-permanent-molars showed a higher incidence of an increase in severity of TFI score.

## Background

Dental fluorosis (DF) is the result of a subsurface hypomineralization of the enamel due to chronic and cumulative duration of excessive fluoride (F) ingestion during amelogénesis [1, 2]. Its extent and severity have been related to the amount and timing of F intake [3]. Teeth are more susceptible to DF when they are in the early maturation stage of development [4]. Additionally, since dental formation occurs by incremental stages and the development of each tooth occurs at different times, the period of maximum susceptibility for each tooth and each area also varies [2, 5].

Its clinical appearance varies from white diffuse and irregular lines in mild cases to loss of structure and marked discoloration in the more severe forms [1]. Teeth with DF higher severity score show decreased resistance to demineralization, compared to sound enamel on lab studies or natural teeth [6].

Although there are few longitudinal studies and follow-up reports published regarding DF comparing to dental caries, some authors have reported that there seems to be no association between both entities [7–9]. There is little evidence about DF clinical changes over time and their natural history. Most reports are related to fluoride intake, and those that report change use different indices to evaluated DF. In 1988, de Liefde [10], reported an increased score to diffuse opacities in children 9–12 years of age by using the developmental defects of enamel (DDE) Index. In 2016, Wong et al. [11] applied the DDE Index and reported a decrease in the severity of diffuse opacities. Also, in 2016, Do et al. [8] observed that very mild and mild lesions of DF decreased in severity over time, using the TFI Index.

In vitro and descriptive studies have reported the disappearance of mild DF lesions on the incisal edges due to wear and occlusal surfaces exposed to attrition [1], while severe lesions with higher enamel porosity evidence greater fragility at the outer surface, which can fracture easily with mechanical chewing forces. In post-eruptive enamel fractures, the surface is lost at the cusps and incisal edges, becoming more severe, and the general form of the tooth may be affected [1, 12].

Structural damage can increase in the long term depending on severity [13, 14]. Although the appearance of minor lesions is generally accepted socially, moderate and severe forms can sometimes compromise aesthetics and generate treatment needs in individuals and concerns regarding the impact on the quality of life [11, 15–17].

The available evidence is limited, and it is not yet clear whether DF lesions follow a defined pattern as to whether its severity increases or decreases post-eruption. Additionally, studies using specific DF indexes could provide evidence in understanding clinical change and contribute to establishing guidelines and protocols for the clinical management of DF. This study aimed to describe clinical changes of DF lesions over three years and determine their association with gender, age, and degree of severity in children between 8 and 12 years of age.

## Methods

### Study design and ethical aspects

Two linked cross-sectional evaluations, one in 2015 and one in 2018, were compared using a panel design. The Institutional Ethics Committee for Human Research at CES University approved this study (no.:110-code-718). All participants signed informed consent. This study conforms to the STROBE guidelines for observational studies.

### Settings and participants

El Cedro community is part of the Ayapel municipality and is located in northern Colombia (IDH-M: 0.705). Colombia has fluoridation of cooking salt (180–220 ppm), as a public health program. El Cedro is an isolated rural community that lacks healthcare infrastructure and has limited access by the river for most of the year, with an estimated population of 929 inhabitants and a density of 6.15 inhabitants/km^2^. This study is part of a community-based oral health preventive program, with dental caries follow-up since 2009 [18], and in 2015 began DF evaluation as another variable.

**Fig. 1:**
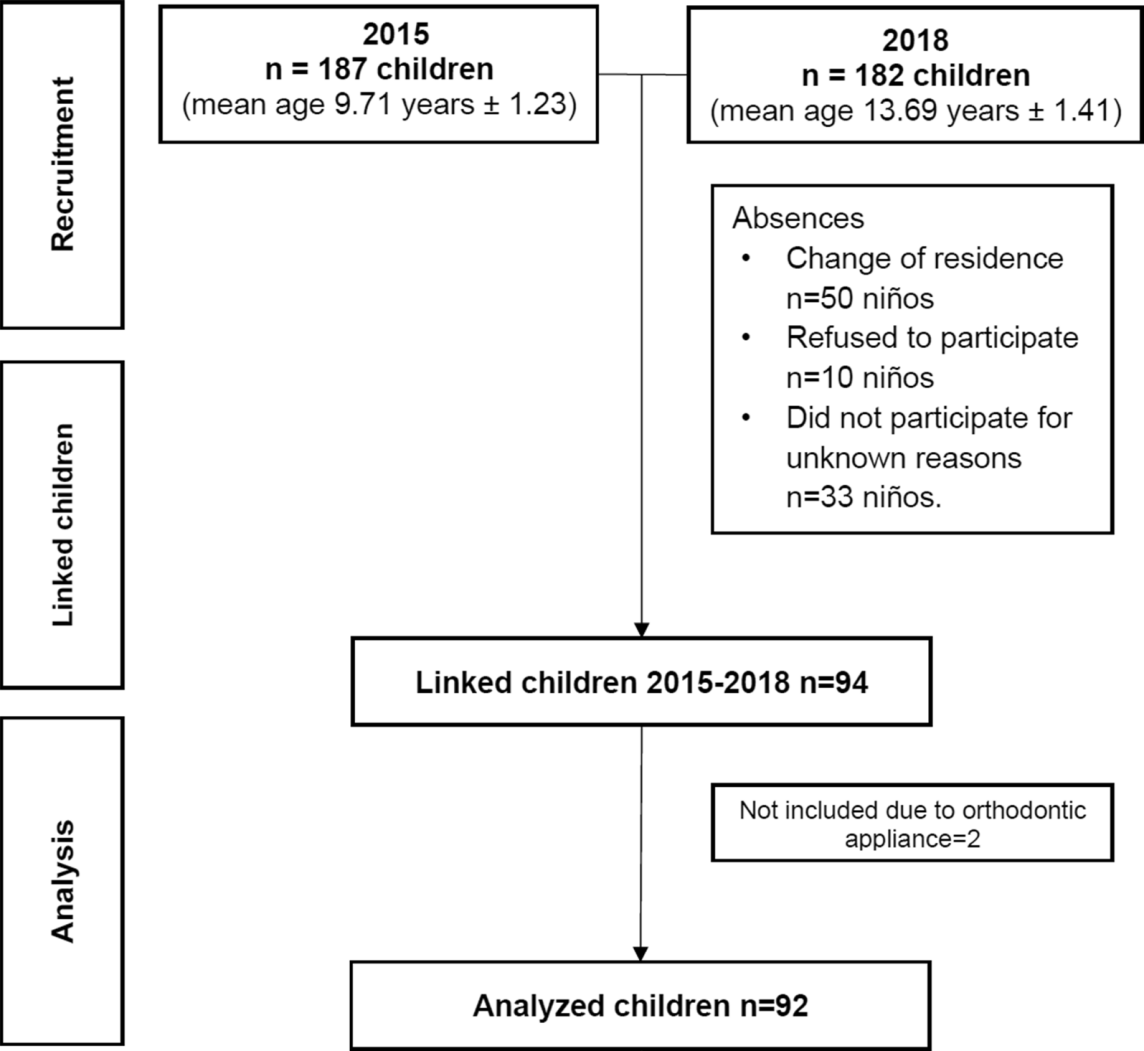
Study flow diagram

In 2015, all children living area and registered in the local school were invited to compose the sample (n = 187; 8–12-years-old); the clinical exam was carried out [19] in all of them. Children included in this evaluation had at least all the permanent first molars and permanent incisors. To evaluate changes in DF lesions after three years, a deterministic linkage was made between both database 2015 (n = 187) and 2018 (n = 182), which included the same children based on name, age, and sex, which totaled 94 individuals, out of which two were excluded due to orthodontic treatment, and so, the teeth of 92 children were compared and analyzed. All children, including those who had previously been evaluated in 2015 were called to a clinical evaluation during four consecutive days. A flowchart is presented in Fig. 1.

### Variables and calibration of the examiners

Clinical evaluations were carried out in a dental office equipped with artificial light, suction-system, water, and air, using a mouth mirror and a periodontal probe recommended by the World Health Organization (WHO) [20]. After cleaning and drying, all the permanent teeth of each individual were evaluated by two previously trained, standardized, and blinded examiners (kappa Intra examiner = 0.89 and Kappa inter examiner = 0.87), according to 10-points Thystrup and Fejerskov [21] Index (TFI) (1978) for DF diagnosis; appropriate differentiation from other opacities and caries white spot lesions, based on Seow's criteria [22] (1997) were made. All permanent teeth were classified according to their maximum TFI score. The same examiners evaluated in 2015 and 2018.

### Statistical analysis

The data was stored, cleaned and processed in the Stata 16^®^ (College Station, TX). For the descriptive analysis, frequencies and percentages were used for categorical variables, and central tendency and dispersion measures were used for age. Contingency tables were constructed to characterize the change in clinical presentation, according to the TFI in all teeth, after three years.

Analysis of associated factors was carried out by classifying change dichotomously as an increase or decrease of the TFI in at least one point [21]. Gender, age, TFI at the first exam, and tooth type were considered as adjustment covariates.

The analysis of factors associated with changes in the clinical presentation of DF was performed by log-binomial regression with robust estimation of variance to account for between-teeth within-child autocorrelation. Results are presented as observed (simple regression models) and adjusted (multiple regression model) Risk Ratios (RR) with 95% confidence intervals and p values. All variables were included for adjusted estimation.

Post-hoc analysis established that the study's sample size had a statistical power greater than or equal to 80% for RR, greater than or equal to 1.1 or less than or equal to 0.9.

## Results

All the permanent teeth of the 92 linked children were evaluated, and their maxim TFI was recorded; the sample had a mean age at the onset of the study of 9.71 ± 1.2 years and 13.7 ± 1.4 years old in the second evaluation. About 53.2% were females, and 97.8% of the children had at least one tooth with DF in the first evaluation.

From a total of 1663 teeth present in the first evaluation that were also examined in the second evaluation, 1117 teeth (67.1%) showed a TFI score to DF ≥ 1 at the initial assessment and were reevaluated.

### Distribution of dental fluorosis according to TFI criteria

**Fig. 2:**
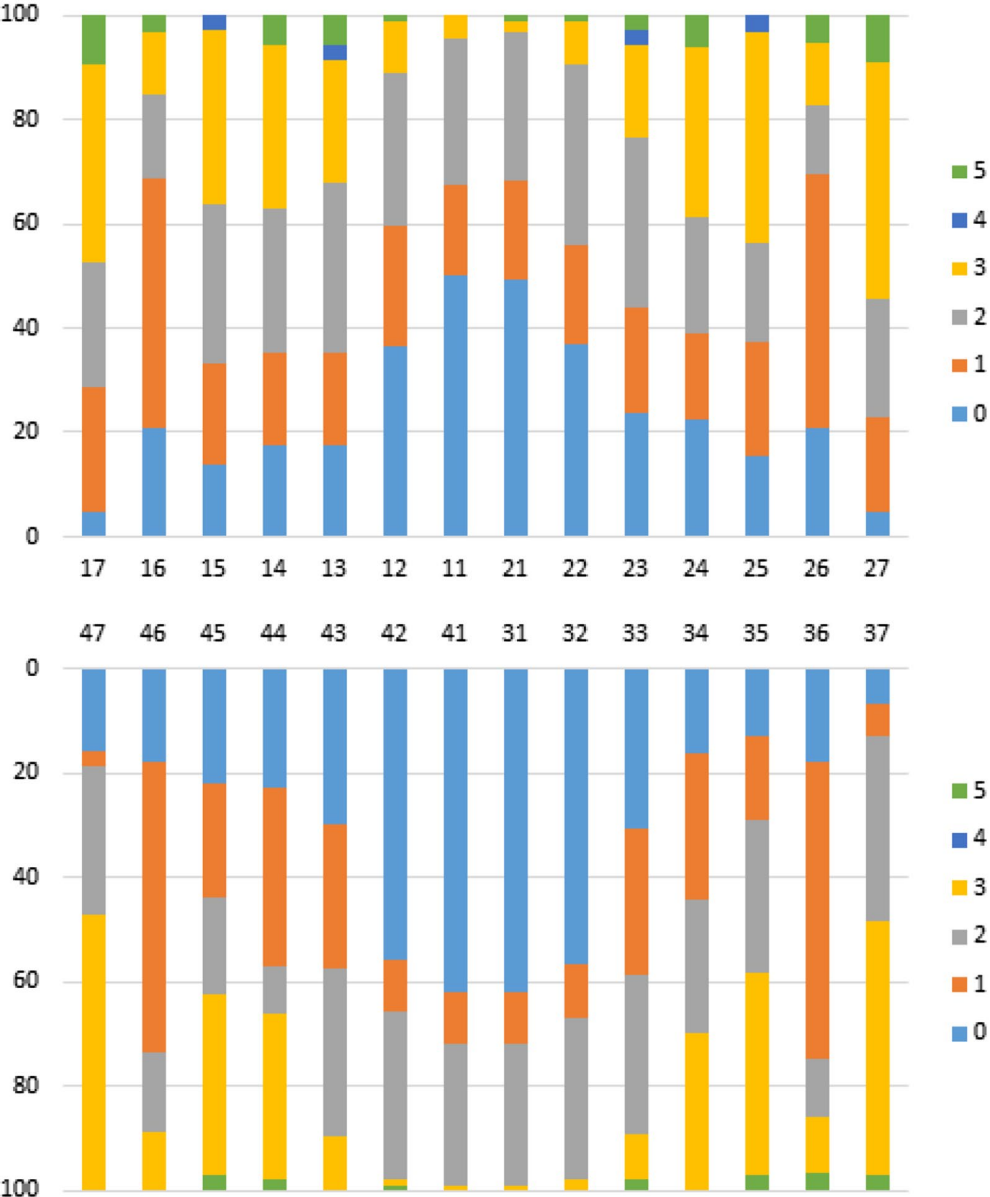
Distribution of TFI scores by tooth at first evaluation

At first evaluation, DF distribution by tooth was homogeneous for both maxillary arches. TF 1 was the most frequent at the first permanent molars (48.9%-to-57.1%), TF 2 was most frequent at the lateral incisors and canines (29.3%-to-34.5%), TF 3 was the most frequent at the second permanent molars (38.1%-to-53.1%), followed by premolars (30.2%-to-40.6%), TF 4 score was only found in canines and second upper premolar (2.8%-to-3.1%), and TF 5 score was more frequent at second permanent upper molars (9.1%-to-9.5%); it was not found severity TF 6–9 scores, at first evaluation (Fig. 2).

### Clinical changes in dental fluorosis, according to TFI criteria

Severity distribution according to the TFI at baseline and after three years is observed in Table 1. A total of 1.117 teeth with a TF ≥ 1 at first evaluation were linked and analyzed after three years (same teeth). About 46.2% of the teeth remained stable; 29.6% of the teeth presented lower TFI scores, and 24.0% of the teeth presented higher TFI scores (Table 1).

**Table 1:** Clinical aspects after 3-years of follow-up, comparing to baseline TFI score

TFI score at baseline		Three-year changes in TFI score		
TFI ≥ 1	Teeth	Lower	Equal	Higher
	n	n (%)	n (%)	n (%)
TF 1	416	47 (11.3)	185 (44.5)	184 (44.2)
TF 2	416	150 (36.1)	213 (51.2)	53 (12.7)
TF 3	248	102 (41.2)	116 (46.8)	30 (12)
TF 4	4	4 (100.0)	–	–
TF 5	33	28 (84.8)	3 (9.1)	2 (6.1)
Total	1117	331	517	269

Frequencies (n) and percentages (%)

About TFI score after three years of follow-up, from those teeth that had an initial TF 1, 11.3% showed lower score (TF = 0) and 44.2% (TF 2 = 39.2%; TF 3 = 5.0%) had higher score. From the teeth that were TF 2 36.1% presented lower (TF 0 = 2.2%; TF 1 = 33.9%) and higher (TF 3 = 12.7%) score. More than one third of the teeth that had an initial TF 3 presented a lower TFI (TF 0 = 0.8%; TF 1 = 8.9%; TF 2 = 31.5%) score, and only 12.0% greater TFI (TF 4 = 5.2%; TF 5 = 6.0%; TF 6 = 0.8%). Four teeth that started in TF 4 diminish to TF 3 (100.0%), and most of the teeth (84.8%) that began with a TF 5 had lower TFI score (TF 0 = 3.0%; TF 1 = 12.1%; TF 2 = 36.4%; TF 3 = 33.3%) and only two teeth (6.1%) increase from TF 5 to TF 6 score (Table 1).

### Factors associated with TFI changes

Multivariate analysis showed a higher incidence of increased scores after three years in males (27.1%) than in females (21.6%), but the difference was not statistically significant RR = 1.4; 95% CI 0.9–2.1 (p-value > 0.05).

Regarding the TFI score at the first evaluation, TF 1 was correlated with greater increase in TF, RR = 9.7; 95% CI 1.7–56.5 (p-value = 0.01). According to the tooth type, when compared with maxillary incisor as a group, the canines-premolars-and second-permanent-molar group of teeth presented a higher incidence of an increase in severity of TF score, RR = 3.3; 95% CI 1.9–5.6 (p-value = 0.005) (Table 2).

**Table 2:** Factors associated with the increase in TFI score in permanent teeth after 3 years

Baseline variable	Incidence	Crude				Adjusted			
		RR	95%IC		p-value	RR	IC95%		p-value
*Sex*									
Male	27.1	1.3	0.8	1.9	0.308	1.4	0.9	2.1	0.093
Female	21.6	1.0				1.0			
*Age*									
8	30.3	1.4	0.7	2.7	0.379	1.6	0.8	3.3	0.165
9	35.3	1.6	0.8	3.0	0.163	1.8	1.0	3.5	0.064
10	37.7	1.7	0.9	3.2	0.106	1.8	1.0	3.4	0.066
11	38.5	1.7	0.9	3.3	0.089	1.7	0.9	3.3	0.082
12	22.2	1.0				1.0			
*Baseline TFI*									
TF 1	44.2	7.3	1.3	40.1	0.022	9.7	1.7	56.5	0.011
TF 2	12.7	2.1	0.4	11.6	0.394	2.8	0.5	16.5	0.251
TF 3	12.1	2.0	0.5	8.1	0.334	1.9	0.4	8.3	0.397
TF 5	6.1	1.0				1.0			
*Tooth type*									
Incisors-primer molar	20.0	1.8	1.0	3.3	0.054	1.1	0.8	1.4	0.678
Canines-premolar-second molar	33.2	3.0	1.7	5.2	0.000	1.5	1.1	2.0	0.005
Maxillary incisors	11.2	1.0				1.0			

**Fig. 3:**
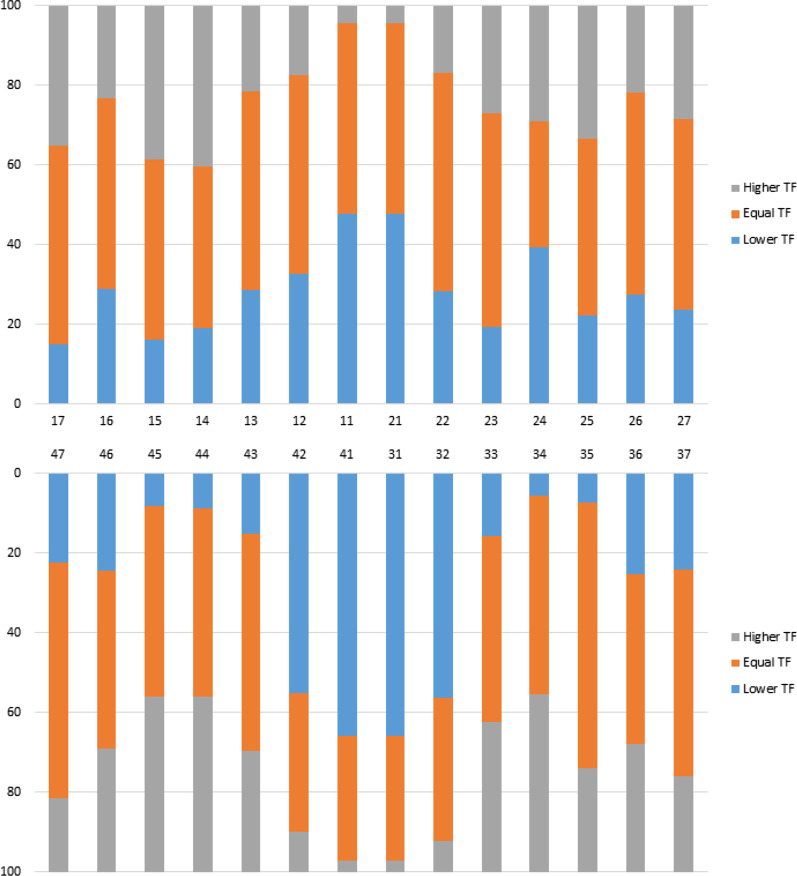
Distribution of TFI score change by tooth after three years

Figure 3 illustrates the percentages of clinical changes in TFI scores by tooth over three years.

In the first-permanent-molars and permanent incisor as a group of teeth, the lower central incisors (66.0%) presented the most frequent decrease of the TFI score, followed by the lower lateral incisors (56.0%). Central incisors showed a higher percentage (48.0%) of teeth between the maxillary incisors with change to a lower TFI score.

Severity changes in the canines-premolars and second-permanent-molars teeth group showed a higher frequency of changes to a higher TFI score in the upper arch in the first right premolar (40.0%), followed by second right premolar (39.0%) and second right permanent molar (35.0%); and in the lower arch, were both right premolars (44.0%) and the first left premolar (44.0%), showed a greater increase (Fig. 3).

## Discussion

This study provides evidence regarding the clinical change of dental fluorosis and its severity after three years, using the TF index for DF. This is the first study to report dental fluorosis clinical change and its relationship with age, gender, and severity in a specific Colombian population with a high prevalence of DF.

The Thylstrup and Fejerskov Index is more applicable for measuring DF severity in areas with high or low fluoridated areas; it is considered a sensitive method because it allows carefully classify the affected tooth by correlating clinical and histological features. Despite being defined more than 40 years ago, it contributes to avoiding underestimating teeth affected by fluorosis; and is the most suitable for use in the clinical management of DF [21, 23].

The present study found that most DF scores remain stable with time, with a tendency to diminish at tooth level. Reduction in severity was noted in 29.6% and the increase in 24.0% of the teeth, while Do et al. [8] reports a reduction in 10.6% and an increase in only 1.0%. Furthermore, the cases of increased severity were observed in the scores 1, 2 (very mild), 3 (mild), and 5 (moderated), with association to TF 1 score (very mild) (p = 0.01); the canines-premolars-second molars as a group also showed association with increased DF severity (p = 0.005).

Regarding score changes, in general, the TF 1 increased until score 3, TF 2 had the greater tendency to remain the same, and scores 3–5 showed a greater tendency to reduce the score. The present study found severity TFI score, ranged from 1 to 6 after three years, differing to Do et al. [8], who reported a severity from 1 to 3, in children between 8 and 13 years old. Although in both studies, predominated a higher proportion of teeth with the same TFI score, after the following, our study presented more considerable change than the Do et al. [8].

After three years, some DF lesions became more evident. According to Thylstrup and Fejerskov [21], it's possible that a subsurface lesion was protected by an outer surface, previously diagnosed with a mild score. The increased severity of dental fluorosis lesions may be related to some intraoral conditions facilitating enamel mineralization [21]. So, in cases of slightly fluorosed enamel and even in moderate cases, macroscopic changes can be described as restricted to the coronal-incisal half of the crown at a specific time. In populations characterized by a low incidence of DF, teeth frequently exhibit opacities, preferentially located on the cusp tips [24], in contrast with the epidemiological scenery of the analyzed population of the present study.

In some cases, changes in the reduction of the DF score could be due to the influence of mechanical forces such as attrition and abrasion, which can cause wear on hypomineralized porous enamel surfaces as scores 1–3, causing lesions that disappear clinically and expose almost normal underlying enamel. As a result of the above, there might sometimes seem to be a gradual posteruptive regression of the severity of DF milder forms [1]. Even moderate and severe lesions can present fractures that can expose normal underlying enamel, decreasing the score of lesions previously classified as moderate or severe.

In this study, 5.2% (n = 59) of the teeth went from score 1 to 0, in agreement with Do et al. [8], that reported 6.6% (n = 97), but in opposite with Curtis et al. [25], that reported a higher percentage (47.0%-to-54.0%). It is noteworthy that some differences between these studies, as follow-up time and the Index applied. However, it has been reported that DF lesions do not disappear entirely; although clinically they may have a healthy appearance, this is due in most cases to changes in its refraction.

Regarding the tooth group, compared with maxillary incisor, the canines-premolars and second-permanent-molars teeth group presented a higher incidence of an increase in the severity of TFI score RR = 3.3; 95% CI 1.9–5.6 (p-value = 0.005). Probably function and mastication forces could be involved. In some cases, this tooth group was recorded with severe scores compared to the first-permanent-molars and permanent incisor as a group of teeth. To Fejerskov et al. [1], the increase in post-eruptive enamel loss depends on the degree of severity at the time of the eruption. In this study, an association was found between the change and the degree of severity of the lesions at the beginning. Canines, premolars, and second molars most frequently show dental fluorosis and more severe in comparison to a lower incidence and severity in the mandibular incisors and first molars [17, 23, 26]. This finding was also observed in the present study.

Some limitations of this study could be the sample size. Only 92 children examined in the first evaluation could be reassessed after three years, possibly due to accessibility or parents' occupation. Although there were linked to the same teeth after three years, there was not a systematic follow-up.

This study analyzed the DF clinical change, with variables as gender, age, tooth, and DF severity score. Nevertheless, some other variables could be omitted. The strengths of our study are the specific DF index used, and all permanent teeth present in the first exam were included, examiners were calibrated, and children had not received any previous treatment such as tooth whitening; whitening was a variable not controlled in other studies [8, 11, 25].

There is limited evidence to compare the longitudinal results of this study; only one study uses a specific index to DF as the one applied in this study, Do et al. [8], while other studies used non-specific indexes as the DDE Index, with controversial results. In 1988, de Liefde [10] found an increase in enamel opacities, while Wong et al. [11] found a decrease in diffuse opacities; other studies used a non-histological validated Index, and not sensitive enough as Curtis et al. [25] However, all of these studies, including ours, provide evidence of the change in DF, its trend, and the moment in which it occurs [2, 8, 10, 11, 25].

In this study, DF higher score after three years did not show statistical association with age, but it was found greater increase at 10 and 11 years of age, while at 12 years, this percentage was lower, showing a similar decline tendency as Curtis et al. [25] who reports a change in a follow-up longer time and to a greater age than this study, that could be evident in a more extensive sample size study. Therefore, factors such as age at the time of evaluation, time the tooth stays in the mouth, post-eruptive enamel maturation, diet, and oral hygiene habits, and its relationship with higher chewing forces in the lower arch, can influence the variation between studies in TFI scores [3, 23, 27, 28].

The post-eruptive environment can change the clinical appearance of dental fluorosis, which may be different according to age, severity and tooth type as was observed in this study; diagnosing DF and identifying the change in the lesion, knowing that a greater percentage of them remains the same or decreases and lower percentage increases, may be useful to define the most appropriate clinical management option for the fluorosed tooth.

Age and severity also influence the aesthetic perception of DF, and there is greater dissatisfaction when DF is severe compared with mild or very mild injuries; some studies have shown that there is no difference between individuals with DF and without DF in their aesthetic perception [23, 29, 30].

It is important higlited that several factors can influence the DF clinical change; in this study, the highest or severe TFI score was assigned to each tooth, which was compared after three years. In each tooth, the score could decrease, remain the same or increase and this alteration could occur in the different evaluated teeth in each child. This means that, in the same individual, some teeth could change to a lower, others remained the same and others increased their TFI score [21, 28]. Whether some teeth increase or decrease in the same individual depends of intrinsic and extrinsic factors, which in each individual can vary and affect clinical appearance and histology, in one or the other tooth following a different pattern between teeth and between individuals.

To better understand the longitudinal clinical change of DF, authors recommend carrying out new studies that allow comparison and approach to knowledge, with longer clinical follow-up. It's important to correlate the clinical diagnosis with the histological component, considering the enamel porosity degree in-depth and extension, using new technologies in clinical diagnosis. Moreover, to identify risk factors and use specific measurement systems for DF, such as the one used in this study based on histological validation. To establish damage prevention measures and inform the professional in clinical evolution.

DF has been one of the most prevalent conditions seen in pediatric dental practice, and it is proper of the clinical professional to guide the patient avoiding unnecessary and invasive treatments.

## Conclusion

The present study based on clinical features about DF confirms the dynamic post-eruptive nature of this condition. After three years of follow-up, a considerable proportion of the teeth changed to a higher score. Furthermore, the canines-premolars and second-permanent-molars teeth group showed a higher clinical change in the TFI score with an increase in severity. The pediatric dentist should include post-eruptive clinical change of DF and feedback from children when making decisions in DF treatment in terms of patient benefit. Record and reevaluate DF-affected teeth over time can impact or change patient perception and treatment needs.

## Data Availability

The data used in the analysis during the current study can be provided by the corresponding author upon request.

## Abbreviations

DF: Dental fluorosis
F: Fluoride
TFI: Thylstrup and Fejerskov Index
RR: Risk rations
CI: Confidence interval
DDE: Developmental defects of enamel
